# Supplementary material for: Integrated Metabolomic and Transcriptomic Analysis Reveals That Amino Acid Biosynthesis May Determine Differences in Cold-Tolerant and Cold-Sensitive Tea Cultivars
Source: Int J Mol Sci. 2023 Jan 18;24(3):1907. doi: 10.3390/ijms24031907 (PMC9916234; doi:10.3390/ijms24031907)
Supplement: Supplementary file 1 [file ijms-24-01907-s001.zip › Supplementary figures.pdf]

Attached Figures

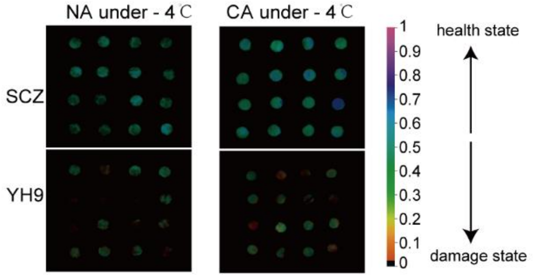

Supplementary Figure S1. Pseudo color image of the photochemical efficiency of photosystem II (Fv/Fm) of SCZ and YH9 tissue culture seedlings subjected to freezing (-4℃,12 h) at NA and CA stages to assess their cold stress resistance. Purple and blue colors indicate the normal state of photosynthetic system, whereas green and yellow indicate the damage to photosystem II.

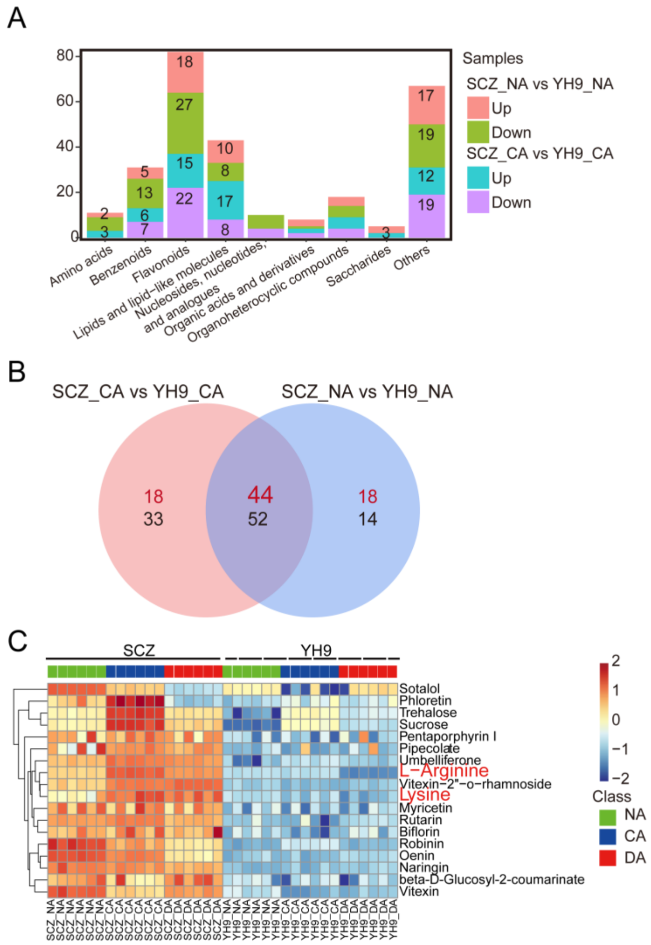

Supplementary Figure S2. Further analysis of differential accumulation metabolites in SCZ_NA vs YH9_NA and SCZ_CA vs YH9_CA. (A) Type and quantity of differential accumulation metabolites. (B) Venn diagrams of differential accumulated metabolites between SCZ and YH9. Red represents up-regulation and black represents down-regulation. (C) Heat map of differential accumulation

metabolites.

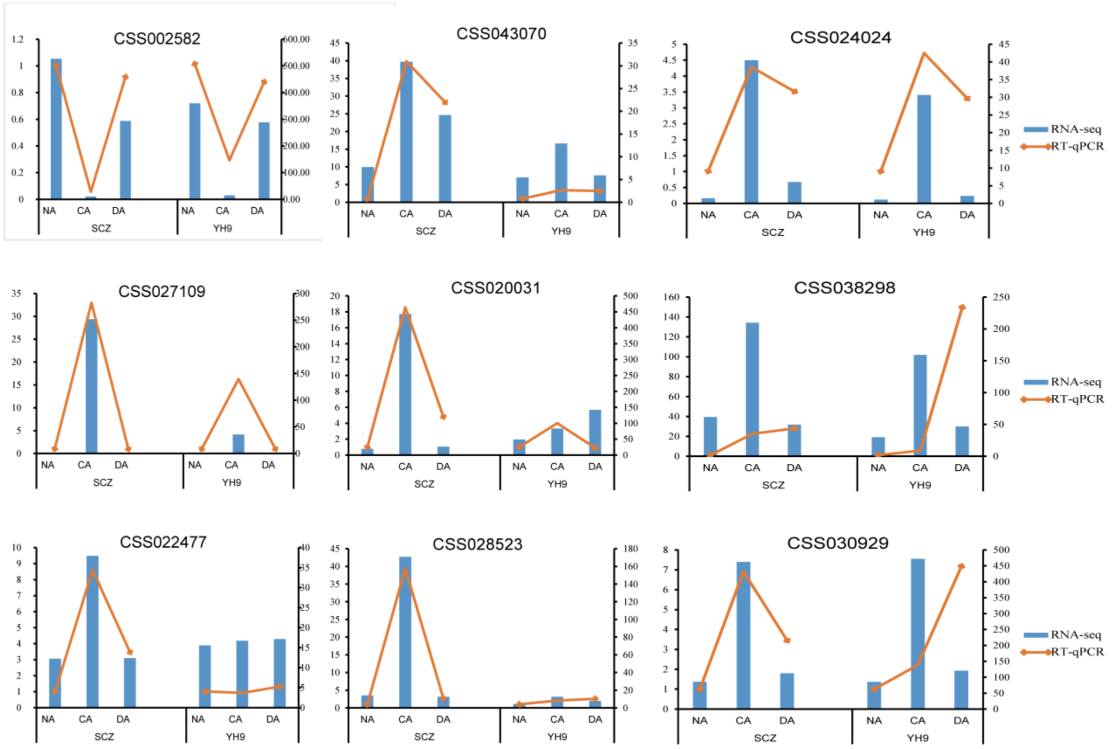

Supplementary Figure S3. Validation of expression patterns of selected differentially expressed genes via RT-qPCR.
